# YY1-dependent transcriptional regulation manifests at the morula stage

**DOI:** 10.17912/micropub.biology.001108

**Published:** 2024-01-16

**Authors:** Mizuki Sakamoto, Takashi Ishiuchi

**Affiliations:** 1 Faculty of Life and Environmental Sciences, University of Yamanashi, Kofu, Yamanashi, Japan

## Abstract

YY1 plays multifaceted roles in various cell types. We recently reported that YY1 regulates nucleosome organization in early mouse embryos. However, despite the impaired nucleosome organization in the absence of YY1, the transcriptome was minimally affected in eight-cell embryos. We then hypothesized that YY1 might prepare a chromatin environment to regulate gene expression at later stages. To test this possibility, we performed a transcriptome analysis at the morula stage. We found that a substantial number of genes are aberrantly expressed in the absence of YY1. Furthermore, our analysis revealed that YY1 is required for the transcription of LINE-1 retrotransposons.

**Figure 1. YY1 regulates transcription at the morula stage.**
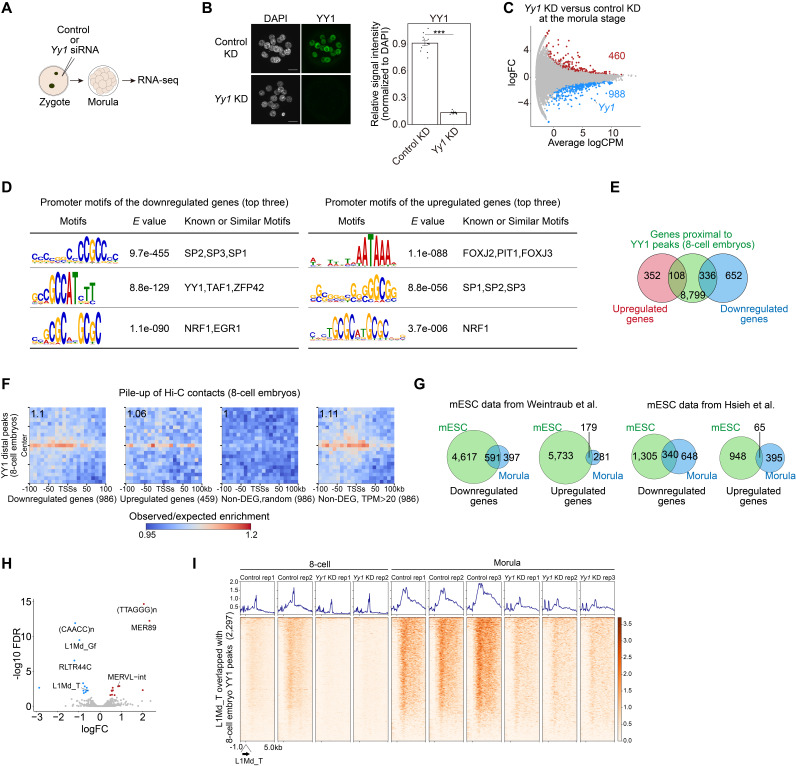
**(A)** Schematic illustration showing the experiments for *Yy1* knockdown (KD). **(B)** (Left) Representative images of immunostaining for YY1 upon *Yy1* KD. Morulae were used for immunostaining. Scale bar, 20 μm. (Right) Plots showing YY1 signal intensity in morulae. Each dot represents the nuclear YY1 signal intensity normalized by the signal intensity of DAPI. ***p < 0.001, Wilcoxon rank sum exact test. Data shown are mean ± s.e.m. n=12 (control KD), 11 ( *Yy1* KD). **(C)** MA plots showing the changes in gene expression between control and *Yy1* KD morulae. Differentially expressed genes were defined by FDR < 0.05. Upregulated (n = 460) and downregulated (n = 988) genes in *Yy* 1 KD embryos are colored in red and blue, respectively. **(D)** Motif analysis at the promoters of downregulated (left) and upregulated genes (right) upon *Yy1* KD. The top three motifs are shown. **(E)** Venn diagrams showing the overlap of between the differentially expressed genes in *Yy1* KD morulae and the genes proximal to YY1 binding sites. **(F)** Pile-up analysis of Hi-C data from eight-cell embryos. (Left two diagrams) Heatmaps showing interaction between YY1-binding sites and the promoters of differentially expressed genes in *Yy1* KD morulae. (Right two diagrams) The same analysis was performed for the promoters of randomly selected or highly expressed non-differentially expressed genes. Genes located on the Y chromosome were excluded. **(G)** Venn diagrams showing the overlap of down- or up-regulated genes in YY1-depleted morulae and mESC. The results from two independent public RNA-seq datasets (Weintraub et al. 2017; Hsieh et al. 2022) are shown on the left and right, respectively. **(H)** Volcano plots showing the changes in the expression of repetitive elements upon *Yy1* KD in morulae. Upregulated (n = 12) and downregulated (n = 12) repetitive elements in *Yy* 1 KD embryos are colored in red and blue, respectively. **(I)** Heatmaps showing the enrichment of RNA-seq reads at YY1-bound L1Md_T loci in control and *Yy1* KD embryos at the eight-cell and morula stages. Multi-mapped reads were included for this analysis.

## Description

YY1, a zinc finger containing transcription factor, is ubiquitously expressed in various cell types and positively or negatively regulates transcription in a context-dependent manner (Gordon et al. 2006; Verheul et al. 2020) . YY1 was also identified as a key structural regulator of enhancer-promoter interactions (Weintraub et al. 2017) , although a recent study showed that enhancer-promoter loop formations were little affected upon the acute depletion of YY1 protein (Hsieh et al. 2022) . YY1 can bind to 5’ UTR of LINE-1 retrotransposons (Minakami et al. 1992; Kurose et al. 1995; Becker et al. 1993; Athanikar 2004; Lee et al. 2010) , and those LINE-1 elements bound by YY1 functioned as enhancers in mouse embryonic stem cells (mESCs) (Meng et al. 2023) . All these studies suggest that YY1 is involved in the regulation of enhancer activities.

We have recently demonstrated that YY1 binds to H3K27ac-marked putative enhancers and regulates nucleosome positioning around these regions in mouse embryos at the eight-cell stage (Sakamoto et al. 2023) . Furthermore, YY1-depleted embryos, generated by injecting siRNA to fertilized eggs, showed a clear developmental delay at the morula-to-blastocyst transition and strongly reduced the birth rate. Thus, zygotically expressed YY1 was necessary for development. However, the depletion of YY1 had a minimal effect on the transcriptome at the eight-cell stage, similar to what was observed upon acute depletion of YY1 protein in mESCs (Hsieh et al. 2022) . However, as developmental defects were evident after the morula formation, we hypothesized that the absence of YY1 for a longer period may display a greater impact on gene expression. To test this hypothesis, we performed RNA-seq at the morula stage, where *Yy1* knockdown (KD) embryos develop normally without developmental delay. We collected *Yy1* KD embryos at the morula stages and performed RNA-seq as has been previously done for eight-cell embryos (Sakamoto et al. 2023) ( Figure 1A ). An efficient depletion of YY1 was confirmed by immunofluorescence at the morula stage ( Figure 1B ). Through transcriptome analyses, we identified 460 upregulated and 988 downregulated differentially expressed genes (false discovery rate < 0.05), which is in sharp contrast to our previous observation that only a few genes were differentially expressed at the eight-cell stage ( Figure 1C, Supplemental Table S 1). Motif analysis of the promoter regions of the differentially expressed genes revealed that the YY1-binding motif was enriched at those of downregulated genes, while such a YY1 motif enrichment was not observed for upregulated genes, suggesting that YY1 positively regulates gene expression in morulae ( Figure 1D ). To further investigate the relationship between YY1 genomic binding and transcriptional alterations, we first extracted the genes proximal to YY1 CUT&RUN peaks (CUT&RUN data from eight-cell embryos) and addressed whether the expression of these genes is affected. This analysis revealed that, among these genes, a larger number of genes were found to be downregulated (336 and 108 genes corresponded to downregulated and upregulated genes, respectively) ( Figure 1E ). Collectively, these results indicate that changes in transcriptome upon YY1 depletion become evident at the morula stage and that YY1 mainly functions as a transcriptional activator at this developmental stage.

We previously found that a large fraction of YY1 binding sites are located promoter-distal regions marked by H3K27ac, which suggested that YY1 binds to active enhancers (Sakamoto et al. 2023) . In addition, the role of YY1 in enhancer-promoter interactions has been suggested. Therefore, we speculated that transcriptional alteration upon *Yy1* KD might be due to some effect on enhancer-promoter interactions. To explore this possibility, using published Hi-C data at the eight-cell stage (Du et al. 2017) , we conducted a pile-up analysis between the promoter-distal YY1-binding sites (data from eight-cell embryos) and the promoters of differentially expressed genes ( Figure 1F ). We found that the distal YY1-binding sites tend to preferentially contact with the promoters of downregulated genes compared to upregulated genes. On the other hand, there were almost no contacts with randomly selected non-differentially expressed genes. However, we noted that the promoters of relatively highly expressed non-differentially expressed genes (TPM > 20) also showed active contacts with distal YY1-binding sites at similar levels to what was observed from the downregulated genes. Thus, YY1-binding sites appear to be in contact with the promoter of active genes already at the eight-cell stage, and a fraction of these genes are downregulated upon YY1 depletion at the morula stage but not at the eight-cell stage. Although the precise reason of the temporal changes in YY1 dependency for transcriptional regulation is not yet clear, the continuous absence of YY1 from its target enhancers may severely affect the enhancer function, which is reminiscent of what was observed in mESCs (Hsieh et al. 2022) .

To address whether the YY1-mediated transcriptional regulatory mechanism in morulae can be similarly observed in mESCs, we compared our transcriptome datasets with two public RNA-seq datasets from mESCs (Weintraub et al. 2017; Hsieh et al. 2022) . Notably, a large fraction of downregulated genes in morulae were also downregulated upon YY1 depletion in mESCs, indicating that the expression dependency on YY1 established around the morula stage is maintained in cells at subsequent developmental stages ( Figure 1G ).

LINE-1 retrotransposons contain the YY1-binding motif at their 5’ UTR, and YY1 binding to LINE-1 is required for transcriptional initiation of LINE-1 (Minakami et al. 1992; Kurose et al. 1995; Becker et al. 1993; Athanikar 2004; Meng et al. 2023; Lee et al. 2010) . To investigate the effect of YY1 depletion on LINE-1 expression, we analyzed changes in the expression of repetitive elements and found that LINE-1 subfamilies such as L1Md_Gf and L1Md_T were significantly downregulated ( Figure 1H ). As a higher number of YY1-binding peaks were observed at L1Md_T loci compared to L1Md_Gf (2,927 and 222, respectively), we further investigated the influence of YY1 depletion on L1Md_T expression. L1Md_T elements were transcribed in both control eight-cell embryos and morulae and underwent transcriptional upregulation during the transition from the eight-cell stage to the morula stage. In contrast, the transcription of L1Md_T was severely suppressed upon YY1 depletion at both stages ( Figure 1I ). Given that LINE-1 activation after fertilization plays an essential role in embryonic development (Jachowicz et al. 2017; Li et al. 2022; Percharde et al. 2018) , this impaired LINE-1 transcription might be linked to developmental defects observed in YY1-depleted embryos.

In this study, we showed that YY1 is required for transcriptional regulation of a set of genes and repetitive elements at the morula stage. However, it is still unclear why YY1 depletion causes impaired gene expression at the morula stage but not at the eight-cell stage. Our data suggest that YY1 binding itself might not directly affect transcription. Rather, YY1 might function to organize chromatin environment or nucleosome positioning at its target enhancers and permit the recruitment of other transcriptional activators. It would be required to examine what transcription factors or chromatin regulators cooperatively function with YY1 to control transcriptional dynamics during early development.

Our results indicated that YY1 promotes the transcription of L1Md_T elements, consistently with previous reports (Athanikar 2004; Lee et al. 2010; Gerdes et al. 2023) . On the other hand, it has been also reported that YY1 represses LINE-1 expression in human embryonic stem cells (hESCs), somatic cells, and mESCs (Sanchez-Luque et al. 2019; Meng et al. 2023) . A recent study showed that 5’ UTR of L1Md_T works as enhancers in mESC (Meng et al. 2023) . Similarly, our data suggested that the YY1-L1Md_T interaction might be involved in gene regulation in addition to L1Md_T transcription in early mouse embryos. Considering the presence of LINE-1 copies throughout the genome and the cell type-specific regulation of LINE-1 by YY1, the YY1-L1Md_T interaction may have a pivotal role to rewire gene expression program.

## Methods

**Animals and collection of mouse oocytes**

All animal experiments were approved by the Animal Experiments Committee of University of Yamanashi (A4-1) and performed according to the guidelines for animal experiments at University of Yamanashi. Female and male BDF1 (C57BL/6N × DBA/2) mice were purchased from SLC. Mice were housed in cages under specific pathogen-free conditions and had free access to water and food. BDF1 female mice (8-12 weeks old) were super-ovulated by injecting 7.5 IU of pregnant mature serum gonadotropin (PMSG), followed by injection of 7.5 IU of human chorionic gonadotropin (hCG) 46-48 h later.

**In vitro fertilization and siRNA injection**

For in vitro fertilization, spermatozoa were obtained from BDF1 male mice (10-12 weeks old). For capacitation, the spermatozoa were cultured in human tubal fluid (HTF) medium for 1 h before insemination. Cumulus-oocyte complexes were obtained from super-ovulated BDF1 female mice and inseminated with capacitated sperm in HTF medium. At 1-2 h postinsemination (hpi), the zygotes were washed and cultured in EmbryoMax KSOM medium (Merck Millipore). For *Yy1* knockdown, a silencer select siRNA (10 µM in H _2_ O; Thermo Fisher) was injected into the cytoplasm of zygotes at 2-3 hpi, and pronuclear formation was verified at 6 hpi.

**Immunofluorescence**

Morula embryos were collected at 64 hpi. Embryos were fixed by 4% paraformaldehyde (PFA) in PBS for 10 min at room temperature and then permeabilized using 0.2% Triton X-100 (Tx) in PBS for 10 min at room temperature. The fixed embryos were washed with PBS containing 0.02% Tx three times and incubated in a blocking buffer (3% BSA in PBS) at 4 °C overnight. Embryos were incubated with YY1 antibody (Santa Cruz Biotechnology sc-7341, 1:500) at 4 °C overnight. Embryos were then washed with PBS containing 0.02% Tx for three times and incubated with anti-mouse IgG labeled with Alexa Fluor 488 (Thermo Fisher A-11001, 1:500) for 1 h at room temperature. The embryos were then mounted on Vectashield (Vector Laboratories) containing DAPI (4′,6-diamidino-2-phenilindole). Fluorescence signals were detected using confocal microscopes FV1200 (Olympus). For quantitative analysis, fluorescence signal intensity was measured using Fiji software (Schindelin et al. 2012) . YY1 signal in each nucleus was normalized by DAPI signal. Statistical analysis was performed using R (version 4.0.3).

**RNA-seq**

Morulae were collected at 64 hpi, washed with PBS containing 0.1% BSA for three times, flash frozen with liquid nitrogen and stored at -80 °C until use. Each replicate contained ten morulae. SMART-Seq stranded kit (TAKARA) was used to prepare RNA-seq libraries. The first and second amplification steps were carried out for 10 and 13 cycles, respectively. Paired-end sequencing was performed on an Illumina NovaSeq X (150 bp ×2).

**RNA-seq data processing**

For samples prepared using a SMART-Seq Stranded Kit (Takara), the first three bases of Read 2 derived from the SMART-seq Stranded Adaptor were removed using Trim Galore! before aligning to the mouse genome (mm10) with Hisat2 (version 2.2.1) (Kim, Langmead, and Salzberg 2015) . Published RNA-seq data (GSE99521 and GSE178982) were also analyzed. Reads corresponding to ribosomal RNA were removed using a bedtools ‘intersectBed’ function. Read counts were calculated by featureCounts (Liao, Smyth, and Shi 2014) with option ‘-p -O --fraction -s 0’ and the expression of each gene annotated by GENCODE vM25 was quantified by calculating TPM. For quantification of repetitive genomic regions, we used featureCounts with option ‘-M -p -s 0’ to allow for multi-mapped read counts of repetitive loci annotated by RepeatMasker. Repetitive elements with more than 100 copies were analyzed. The resulting read count data were processed using EdgeR (Robinson, McCarthy, and Smyth 2010) to identify differentially expressed genes. A false discovery rate of < 0.05, was used to extract differentially expressed genes. Heatmaps were generated using ‘computeMatrix’ and ‘plotHeatmap’ functions in deepTools (version 3.5.1) (Ramírez et al. 2016).

**Motif analysis**

MEME-ChIP (version 5.5.4) (Machanick and Bailey 2011) was used to analyze transcription factor motifs with the following parameters: Classic mode / MOUSE (Mus musculus) DNA/ HOCOMOCO Mouse (v11 CORE) / 2nd order model of sequences / Minimum width: 6 / Maximum width: 15 / Zero or one occurrence per sequence / Number of motif:5.

**Meta-analysis of YY1-binding sites**

YY1 binding peaks in eight-cell embryos were identified in our previous study (Sakamoto et al. 2023) . Genes proximal to these YY1-binding peaks were identified by using annotatePeaks.pl from HOMER based on GENCODE vM 25 (Heinz et al. 2010) . The repetitive elements that overlap with the YY1 binding peaks were identified using bedtools ‘intersect’ function.

**Hi-C data processing**

Hi-C data from mouse eight-cell embryos were downloaded from GSE82185. Hi-C reads were aligned to the mouse genome (mm10) using bwa-mem2 (version 2.2.1) (Vasimuddin et al. 2019) with option ‘-SP5M’ after removing adaptor sequences and low-quality reads by Trim Galore!. The mapped reads were processed to generate Hi-C pairs by pairtools (version 0.3.0) (Abdennur et al. 2023) . The duplicated contact pairs were removed and replicated data were merged by ‘merge’ function in pairtools. Contact matrix were generated at 10-kb bins using cooler (version 0.9.3) (Abdennur and Mirny 2020) and ‘balance’ function in cooler was applied to the output files to correct the bins of Hi-C matrices. For pile-up analyses, we first compiled all possible combinations of YY1 distal peaks and transcriptional start sites of genes of interest on the same chromosome and generated bedpe format files. Then, we conducted pile-up analyses using ‘coolpup.py’ (version 1.1.0) (Flyamer, Illingworth, and Bickmore 2020) with option ‘--features_format bedpe --nshifts 1 --seed 0’ and contact signal was depicted using ‘plotpup.py’ with option ‘--scale linear’.

**Quantification and statistical analysis**

Statistical analyses were implemented with R ( http://www.r-project.org ).

**Data availability**

All sequencing data have been deposited in the Gene Expression Omnibus (GEO) under the accession number GSE252053.

## Extended Data

Description: List of differentially expressed genes between control and Yy1 knockdown morula embryos. Resource Type: Dataset. DOI: 10.22002/s69zk-3m482
